# Generative multimodal large language models in mental health care: Applications, opportunities, and challenges

**DOI:** 10.1371/journal.pmen.0000488

**Published:** 2025-11-06

**Authors:** Ariel Soares Teles, Jaya Chaturvedi, Tao Wang, Marcia Scazufca, Yamiko Msosa, Daniel Stahl, Angus Roberts

**Affiliations:** 1 Department of Biostatistics and Health Informatics, Institute of Psychiatry, Psychology and Neuroscience, King’s College London, London, United Kingdom; 2 Campus Araioses, Federal Institute of Maranhão, Araioses, Maranhão, Brazil; 3 Hospital das Clínicas HCFMUSP, Faculdade de Medicina, Universidade de São Paulo, São Paulo, Brazil; PLOS: Public Library of Science, UNITED KINGDOM OF GREAT BRITAIN AND NORTHERN IRELAND

## Abstract

Generative Large Language Models (LLMs) are transforming mental health care by enabling the generation and understanding of human-like text with increasing nuance and contextual awareness. However, mental health is a complex, multidimensional domain that often requires richer sources of information beyond text. This narrative review explores the emerging role of Multimodal LLMs (MLLMs), which are models that integrate diverse input modalities such as speech, images, video, and physiological signals, to incorporate the multifaceted nature of mental states and human interactions. We first outline the foundational principles of MLLMs and their distinction from traditional text-only LLMs. We then synthesize recent empirical studies and experimental applications of MLLMs in mental health research and clinical settings, highlighting their potential to improve diagnostic accuracy, enable real-time monitoring, and support context-aware, personalized interventions. Finally, we outline opportunities for future research and innovation, and discuss key implementation challenges in MLLM-based mental health care.

## Introduction

Mental health has become an increasingly critical issue worldwide, with rising rates of anxiety, depression, and other mental health disorders exacerbated by factors such as social isolation, economic pressures, and global crises [1]. The growing recognition of mental health’s impact on overall well-being, productivity, and quality of life has underscored the urgent need for effective and accessible care. However, traditional mental health services often face significant barriers, including stigma, high costs, and a shortage of trained professionals, leaving many individuals without adequate support [2]. The mental healthcare sector has already acknowledged the urgency for innovative solutions [3,4]. In response, digital technologies have emerged as a transformative force in addressing these challenges, offering innovative solutions such as mobile applications, teletherapy, chatbots, and virtual reality [5,6]. Such solutions can be further enhanced through the integration of Artificial Intelligence (AI) [7], which enables more responsive, adaptive, and context-aware systems. By harnessing recent advancements in AI, digital technologies hold significant promise for transforming mental health care, expanding access, enhancing personalization, and supporting more effective and scalable interventions.

Large Language Models (LLMs) represent a significant advancement in AI, capable of recognizing, generating, and interacting with human language [8]. These models are trained on vast datasets, enabling them to perform a wide range of tasks, from answering questions and summarizing text to generating creative content and providing personalized recommendations. In healthcare, LLMs have already demonstrated utility [9,10], and hold potential to revolutionize various aspects of care delivery, including diagnostics, patient communication, and clinical decision support [11,12]. For example, they have been investigated as virtual therapists [13] and employed to develop diagnostic and prognostic models based on Electronic Health Records (EHR) data [14]. Therefore, LLMs offer a promising avenue for improving healthcare accessibility, efficiency, and personalization, particularly in areas like mental health care where scalable, personalized, and timely interventions are extremely important.

Developed from 2022 [15], Multimodal LLMs (MLLMs) extend the capabilities of text-only LLMs by integrating and processing more than one data modality, including not only text but also audio, image and video, enabling a more comprehensive understanding of complex inputs [15]. These models are designed to both interpret complex, multimodal inputs and produce outputs in one or more modalities, supporting richer and more flexible interactions. Considerable efforts have been dedicated to creating LLMs that handle multimodal inputs and tasks, ultimately leading to the development of MLLMs [16], which analyse diverse health data to enhance, for example, diagnostics, treatment planning, and personalized care [17]. Therefore, MLLMs represent a significant advancement in healthcare, addressing the multimodal nature of medicine, where handling multiple data types is often required [18,19].

While recent reviews have comprehensively examined the applications, limitations, and ethical implications of text-based LLMs in mental health care and psychiatry [20–29], they have largely focused on unimodal models. These secondary studies are highly relevant and timely, shedding light on key themes such as early screening, conversational agents, and clinical integration. However, none have specifically addressed the emerging class of MLLMs. Our investigation of the existing literature suggests that this is the first review to concentrate on the application of MLLMs in the field of mental health. By examining not only current applications, but also presenting potential areas for future studies, our review contributes a novel perspective on how MLLMs may advance digital mental health solutions.

Although language is a fundamental modality, because human communication is intrinsically linguistic, relying solely on text risks overlooking crucial dimensions of mental health. By integrating additional modalities, MLLMs can offer a more comprehensive and nuanced understanding of mental health. Therefore, the objective of this narrative review is to explore the emerging role of generative MLLMs in mental health care. Specifically, we synthesize recent empirical and conceptual developments on how MLLMs have been integrated and applied within mental health research and clinical contexts, recognizing that most current evidence remains exploratory or proof-of-concept in nature. We begin by outlining the foundational concepts underlying MLLMs and how they differ from text-only LLMs. We then examine empirical studies and experimental systems that demonstrate their use in mental health settings. Finally, we identify opportunities for future applications and derived studies, as well as important challenges associated with model implementation.

## What are generative multimodal LLMs?

A language model is a probabilistic model designed to estimate the likelihood of a sequence of tokens, typically words or subwords, within a given context. Contemporary state-of-the-art language models are predominantly based on the transformer architecture [30], which leverages self-attention mechanisms to capture contextual relationships between tokens across an entire sequence. Transformer-based architectures can be broadly categorized into three classes [31] (Table 1): encoder-only, decoder-only, and encoder-decoder models, each optimized for different types of tasks. Among them, decoder-only and encoder-decoder models are typically considered generative. Decoder-only models are explicitly trained for autoregressive language modelling, where the objective is to predict the next token in a sequence given the preceding tokens. Encoder-decoder models are also generative, particularly in tasks that require transforming an input sequence into a new output sequence.

**Table 1:** Overview of transformer-based language models.

Model Type	Architecture	Typical Input	Output	G	Example Models	M
Encoder-only	Transformer	Text	Text (embedding)	No	BERT [32], RoBERTa [33]	No
Decoder-only	Transformer	Text (prompt)	Text (generated)	Yes	GPT-2, GPT-3 [8]	No
Encoder-decoder	Transformer	Text	Text	Yes	T5 [34], BART [35]	No
**MLLM**	Hybrid (transformer-based)	Text, images, audio, video	Text, image, audio, video	Yes	LLaVA [36], LLaVA-NeXT-Interleave [37], LLaVA-Video [38], GPT-4o [39,40], GPT-5 [41], Gemini [42], Veo 3 [43]	Yes

Note: G = Generative; M = Multimodal

MLLMs are deep learning models that extend the capabilities of text-only language models by incorporating and jointly processing multiple types of input modalities, such as text, images, audio, video, and physiological signals [15]. While unimodal models operate solely on textual data, MLLMs are designed to integrate heterogeneous information streams to build a richer and more holistic representation of the world [44]. This integration enables the models to perform tasks that require reasoning across modalities [45,46]. For example, generating descriptive text from an image, generating a video from a textual description, or interpreting emotional states to some extent from both spoken language and facial expressions.

**Fig 1:**
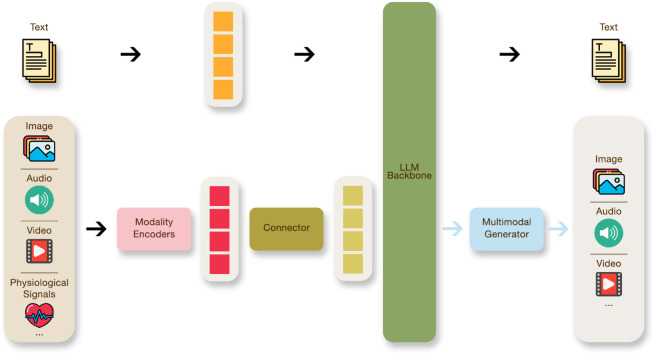
Generic architecture of a Multimodal Large Language Model (MLLM), illustrating how modality-specific encoders, a connector, and an LLM backbone jointly process heterogeneous inputs to generate textual or multimodal outputs.

MLLMs can be understood as layered architectures that extend unimodal LLMs to process heterogeneous data. Conceptually, they can be viewed as layered systems comprising three core modules (Fig 1): a modality encoder, a modality interface (connector), and a language model backbone, with an optional generator for multimodal output synthesis. At the input stage, modality-specific encoders [47] transform raw signals such as images [48], audio [49], video, or physiological data into vector embeddings [50]. These encoders are typically pre-trained models aligned across modalities. They function as the perceptual components of the system, transforming raw multimodal inputs into structured embeddings that effectively capture and preserve semantic information.

Embeddings are then mapped into the token space of the language model through a connector [51], which aligns different modalities with a unified representation space [15,52]. Depending on the fusion granularity, connectors can operate at the token level or at the feature level. This interface ensures that heterogeneous embeddings are projected into a shared latent space that the LLM backbone can understand. By aligning multimodal inputs in this unified representational space, MLLMs enable seamless joint reasoning across different information types. Once aligned, the LLM backbone performs integrated reasoning by attending jointly to textual tokens and projected multimodal embeddings. The fused representation allows the LLM to produce coherent, context-aware responses that integrate insights from all modalities involved. Finally, some architectures attach a multimodal generator to extend output capabilities beyond text. These generators can produce images, audio, or videos, often via diffusion-based or autoregressive decoders [15].

Some MLLMs are primarily designed to generate textual outputs from non-textual inputs, while others extend this capability toward temporal and multimodal reasoning. For instance, LLaVA [36] interprets visual content through a language interface, producing detailed captions and visual question-answering outputs. Building on this foundation, LLaVA-Video [38] introduces instruction-tuning for dynamic video understanding, enabling fine-grained captioning and reasoning over temporally rich video sequences. Similarly, Veo 3 [43] represents a new generation of generative video models that demonstrate zero-shot visual reasoning across perception, modelling, manipulation, and temporal inference tasks. Trained on web-scale video data with a generative objective, Veo 3 can segment, edit, and reason about physical interactions, solve visual puzzles such as mazes and symmetry completion, and exhibit “chain-of-frames” reasoning analogous to chain-of-thought processes in LLMs [53]. In parallel, GPT-4o [39,40] and GPT-5 [41] exemplify a natively multimodal architecture capable of bidirectional generation across text, image, and audio. They can not only generate descriptive text from visual and auditory inputs but also perform tasks like answering spoken questions, analysing images in real time, and reasoning jointly across text, vision, and audio modalities, demonstrating fluency and flexibility in multimodal interaction.

A key distinction lies between “true” MLLMs and unimodal LLMs integrated within multimodal pipelines. True MLLMs are architecturally designed to natively process and generate across multiple modalities through joint embeddings and cross-modal reasoning. In contrast, some systems employ unimodal LLMs as downstream reasoning engines in larger multimodal frameworks. They combine multimodal data streams but pass only structured or textualized representations to a unimodal LLM, which then generates textual outputs. While these pipelines do not constitute MLLMs *per se*, they are important to consider in this review because the data itself is multimodal, and unimodal LLMs act as reasoning engines that translate heterogeneous signals into clinically meaningful, natural-language outputs. Moreover, such systems illustrate the transitional stage in which unimodal LLMs extend into multimodal domains, highlighting both the opportunities and the limitations that motivate the development of true MLLMs. In this review, we consistently use the abbreviation “MLLM” for models with native multimodal architectures, while referring separately to unimodal LLMs in multimodal frameworks.

## MLLMs applications in mental health

MLLMs hold promise across a wide range of mental health applications [54,55]. This section focuses on recent empirical studies and experimental systems that have already leveraged MLLMs in mental health contexts.

### Therapy assistance

Generative LLMs are being explored as tools to assist psychotherapy, both by interacting directly with individuals as virtual therapists and by enhancing the education and training of mental health professionals. For clinicians and trainees, LLMs can serve as accessible educational aids, simulating therapeutic dialogues [56], and offering feedback on clinical communication [57,58]. Simultaneously, LLMs can engage directly with users (i.e., chatbot-based interventions [59]) by delivering responses that emulate therapeutic dialogue [60]. While multimodal capabilities may eventually broaden these applications, current evidence suggests that even text-only models are useful.

Even in the absence of multimodal inputs, LLMs have demonstrated the ability to generate responses that are perceived as empathic, supportive, and aligned with core therapeutic principles. For instance, recent evaluations have shown that licensed clinicians rate AI-generated psychological advice as comparable to (or in some dimensions even more favourable than) expert-authored responses, particularly in terms of emotional and motivational empathy [13,61]. These findings suggest that even without access to multimodal information (e.g., tone of voice, facial expressions), text-based LLMs can simulate therapist-like interactions and generate emotionally resonant messages that, in some evaluations, were rated comparably to those written by professionals.

Recent advances in MLLMs have begun to address the limitations of purely text-based therapeutic systems by integrating visual cues, such as facial expressions and body posture, into the therapeutic dialogue. Zhu *et al*. [62] proposed a framework that enhances mental health support by embedding a unimodal LLM within a multimodal pipeline. The system combines facial expression analysis, dialogue history, commonsense reasoning, and context-specific counselling strategies. Emotional cues are first extracted from visual signals and transformed into textual descriptions, which are then passed to the LLM for response generation. Although the language model itself remains unimodal, the overall framework integrates multimodal information at the system level. Evaluations showed that this pipeline produced responses rated higher in coherence, empathy, and helpfulness compared to text-only baselines, highlighting the value of using multimodal data even when the core model is unimodal.

Wang *et al*. [63] conducted a mixed-methods investigation to explore how MLLMs might be meaningfully integrated into psychotherapy for college students, with input from both therapists and student clients. Their findings suggest that, at this stage, MLLMs are best positioned as auxiliary tools that support, rather than replace, human therapists. Participants across all stakeholder groups highlighted potential applications in structured phases of therapy (e.g., initial information gathering, triage matching, and feedback analysis), where MLLMs could assist by analysing multimodal inputs (e.g., speech, facial expressions, self-report data) and generating summaries or suggestions. Both therapists and students also expressed interest in real-time emotion recognition and personalized support, though concerns about emotional depth, ethical risks, and privacy remain strong.

### Mental health monitoring

A key application of MLLMs is their potential to assist in the early detection and intervention of mental health conditions. These capabilities emerge from their integration into monitoring technologies (e.g., personal sensing [64], digital phenotyping [65]). By analysing data collected from smartphones, wearables, and other ambient sensors (e.g., speech patterns, typing behaviour, facial expressions), LLMs can help identify subtle and context-specific changes that may signal emerging psychological distress. When deployed in conjunction with these sensing systems, LLMs can interpret complex, multimodal behavioural data to flag at-risk individuals, deliver timely feedback, or trigger human-led follow-up. This enables scalable, continuous, and personalized mental health monitoring that moves toward just-in-time adaptive interventions [66].

A few studies have already initiated the integration of unimodal LLMs within multimodal monitoring frameworks. One example of such integration is SereniSens [67], a multimodal AI framework designed to monitor and predict stress levels during sleep by combining physiological sensing and language-based interaction. The system uses biosignals such as heart rate variability, respiration rate, blood oxygen level, and body temperature to classify users into five stress levels, using conventional machine learning algorithms. While the input data is multimodal, the LLM component is unimodal: it receives structured outputs from the predictive models and transforms them into personalized, therapeutic messages. A fine-tuned version of GPT-3.5 Turbo is used to interpret the predicted stress levels and associated metrics, generating context-sensitive dialogue through a chatbot interface. While not a true MLLM, this architecture exemplifies how LLMs can function as natural language reasoning engines in larger sensor-based systems, bridging the gap between low-level physiological signal analysis and high-level, user-facing mental health support. Rather than directly ingesting multimodal inputs, the LLM here operates downstream, making it a powerful tool for translating quantitative sensor data into accessible, empathic guidance.

A similar architecture is employed in the Intelligent Phenotype Analysis Suite (IPAS) [68], a tool designed to streamline the analysis of digital phenotyping data. IPAS supports researchers in extracting and interpreting behavioural and physiological signals (e.g., GPS, accelerometer, text message logs) captured through platforms like Beiwe [69] and RADAR-Base [70]. As in the SereniSens framework, the LLM used in IPAS is not multimodal. The system utilizes program-aided prompting techniques to generate descriptions, visualizations, and predictions based on time-series data, while also enabling natural language explanations of findings. This allows researchers, including those with limited programming experience, to engage with complex behavioural datasets via a chatbot-style interface. IPAS exemplifies a growing class of applications in which unimodal LLMs function as reasoning and communication layers within multimodal pipelines, in which multimodal data are textualized [14].

Although originally developed for hypertension management, the Hyper-DREAM platform [71] offers valuable insights into how MLLMs can support mental health monitoring when integrated with digital phenotyping systems. The platform collects rich multimodal data (e.g., self-reported health information, behavioural patterns, and physiological signals like blood pressure and heart rate), and synthesizes this information into individualized digital phenotypes. A fine-tuned LLM, deployed as a chatbot, interprets these data streams to generate personalized feedback, health education, and emotional support. While the primary focus is cardiovascular care, the system incorporates mental health indicators such as sleep quality, stress, and mood, demonstrating the broader potential of LLMs to process passively collected multimodal data.

### Diagnostic support

Multimodal LLMs can assist in the diagnostic process by analysing diverse data sources alongside clinical text to identify signs of mental health conditions, including complex or ambiguous cases [72,73]. Their ability to integrate heterogeneous data makes them valuable tools for supporting clinicians in early detection and differential diagnosis.

Toto *et al*. [74] introduced AudiBERT, a deep transfer learning framework for depression screening that integrates both textual and audio data from clinical interviews. By combining pre-trained representations from BERT (for textual transcripts), an encoder-only LLM [32], and models such as Wav2Vec and SincNet (for vocal signals), AudiBERT captures both verbal and non-verbal indicators of depression. A dual self-attention mechanism fuses these modalities into a shared representation optimized for classification. Similarly, Ali *et al*. [75] investigated the use of MLLMs for detecting depression and Post Traumatic Stress Disorder (PTSD) through both text and audio data from clinical interviews. Evaluating models like Gemini 1.5 Pro and GPT-4o mini, the authors demonstrate that combining audio and text inputs improves diagnostic performance compared to unimodal approaches. These works illustrate the potential of multimodal solutions to leverage the rich, multimodal nature of human communication for accurate and scalable diagnostic support.

A recent study by Sadeghi *et al*. [76] demonstrated how LLMs can be combined with facial expression data to enhance depression detection using a fully automated, multimodal approach. The proposed system leverages GPT-based models to extract depression-related features from interview transcripts and combines them with visual features such as facial action units and eye gaze derived from video data. By training a regression model to predict PHQ-8 scores, the authors showed that the multimodal approach outperformed text- or video-only baselines on a held-out test set, particularly when supported by speech quality assessment. Notably, text-based features had the highest predictive power, but visual cues provided complementary information that improved robustness, especially under conditions with reduced verbal expressivity. This work highlights the potential of MLLMs to contribute to objective, scalable, and clinically relevant assessments of depression severity, including in settings where access to trained professionals may be limited.

### Empirical evidence landscape

As observed throughout the previous sections, and consistent with findings from other reviews [16,77], the empirical literature on MLLMs in mental health remains in an early stage of development. Most available studies are proof-of-concept or mixed-methods evaluations focused on technical feasibility, user experience, or therapeutic potential rather than validated clinical outcomes. A few works have included structured human evaluations, but large-scale randomized or longitudinal clinical studies are yet to emerge. The current evidence base therefore reflects an exploratory phase of research, where multimodal systems are primarily assessed in simulated or pilot settings. This limited empirical maturity underscores both the novelty of the field and the need for rigorous, clinically validated studies to evaluate effectiveness and safety.

## Opportunities

While current applications of MLLMs in mental health are still in the early stages, they point to a wide range of promising future directions. This section outlines emerging research opportunities where MLLMs could meaningfully expand the reach, effectiveness, and personalization of mental health care.

### Therapist–AI collaboration

A promising opportunity for MLLMs lies in their potential to support therapists [78]. Rather than functioning autonomously, these models can act as reflective partners in post-session analysis. To illustrate this potential, consider a hypothetical scenario in which a psychotherapist providing telehealth care to a patient with chronic emotional dysregulation and interpersonal difficulties begins to notice limited therapeutic progress. To enhance their clinical insight, the therapist discusses with the patient the possibility of involving an AI model to assist in reviewing recorded sessions. With informed consent, the MLLM is granted access to video, audio, and transcript data from therapy sessions, enabling it to generate structured, therapist-facing reports. The model’s output supports the therapist by summarizing the session’s content, identifying which interventions were effective or ineffective, and suggesting targeted actions for future sessions. Rather than replacing the therapist’s judgment, the MLLM serves as a collaborative analytic tool—highlighting patterns across sessions, surfacing overlooked cues, and offering clinical suggestions grounded in best practices. An illustrative post-session report might include the following elements:

- Session summary: key topics discussed, emotional tone, and therapeutic strategies employed;
- Helpful elements: Moments where interventions (e.g., reflective listening, use of silence) elicited engagement or emotional expression;
- Less effective elements: instances where techniques (e.g., cognitive reframing, goal-setting) were met with resistance or disengagement;
- Recommendations: suggestions for revisiting specific themes, adjusting timing or delivery of interventions, or attending to nonverbal cues in subsequent sessions;
- Longitudinal patterns: emerging dynamics across multiple sessions, such as recurring ruptures or points of progress.

This example illustrates how MLLMs can act as ethically integrated collaborators, validated by the patient and accountable to the therapist’s professional judgment. By supporting longitudinal tracking and offering fresh perspectives on therapeutic interactions, these models have the potential to enhance care delivery in complex or stagnant cases, particularly in remote or digitally mediated settings. However, the actual impact of such collaboration remains an open research question. The integration of MLLMs into therapeutic practice represents a promising area for future research, particularly studies that examine whether this collaboration yields measurable clinical benefits and how it is perceived by both patients [79,80] and mental health professionals [81]. Understanding these perspectives is essential to ensure that MLLM-assisted care is not only technically feasible, but also trusted, acceptable, and aligned with ethical standards in real-world practice.

### Supporting personalized interventions

While innovative solutions have already leveraged text-only LLMs for mental health support [20,21,27,82], MLLMs open new frontiers by incorporating diverse data sources. These models hold considerable promise for advancing the next generation of digital mental health interventions [6]. MLLMs can enhance traditional care models by enabling early and low-threshold access to support. By continuously analysing multimodal data streams (e.g., written reflections, mood logs, facial expressions, or biometric signals from wearables), these models can identify subtle shifts in emotional or behavioural states. For example, a young adult using a self-guided mental health app might regularly submit short journal entries and selfies. Over time, the MLLM could detect signs of distress (e.g., negative affect, social withdrawal, disorganized appearance) and respond with tailored, just-in-time interventions [66], such as grounding techniques, cognitive reframing prompts, or a gentle recommendation to contact a support person. In this way, MLLMs function not only as personalized companions but also as early-warning systems, offering scalable and cost-effective support that bridges gaps in traditional care.

### Education and awareness

Education and awareness are critical components of mental health promotion, as they influence how individuals recognize symptoms, seek support, and respond to others experiencing distress. Yet, traditional educational approaches might fail to reach all audiences effectively, especially those with limited health literacy, learning differences, or cultural and linguistic barriers. MLLMs can offer a transformative approach by delivering mental health information through a combination of text, images, audio, and video. This multimodal strategy aligns with how people naturally process and retain information, improving comprehension, engagement, and recall [83–85]. For instance, an MLLM could convert complex content about depression or anxiety into a short, accessible explainer video using a text-to-video generation model [86] (e.g., Veo 3 [43], LLaVA-Video [38]), allowing users to engage with the material in a more intuitive and emotionally resonant way. These adaptive capabilities are especially useful for supporting individuals with different cognitive profiles, sensory impairments, or educational needs. By tailoring information delivery to the learner, MLLMs can help reduce stigma, foster early identification of mental health concerns, and encourage timely help-seeking, ultimately contributing to more informed, empowered, and mentally healthy communities.

### Developing MLLM-based predictive models

A particularly promising application of MLLMs in mental health care is the development of predictive models that leverage multimodal data. While prediction models based on structured or unstructured data alone have shown value, the integration of both modalities using multimodal machine/deep learning has demonstrated superior performance in prediction tasks [87]. Applying fusion strategies (e.g., early fusion, intermediate fusion, late fusion) [88] offers flexible pathways for building robust predictive tools. These strategies enable MLLMs to model cross-modal relationships [89] that are essential in mental health scenarios, such as detecting early signs of relapse, forecasting crisis events, or identifying non-adherence to therapy or treatment. Each fusion strategy offers unique strengths: early fusion supports joint learning from the outset, intermediate fusion preserves modality-specific features before integration, and late fusion combines independent predictions; making the choice of strategy dependent on data characteristics and clinical goals [14]. These capabilities open a promising direction for future work: designing and validating MLLM-based predictive systems tailored to mental health care.

### Specializing MLLMs for mental health tasks

Another important opportunity for expanding the clinical utility of MLLMs lies in the ability to adapt them to specific mental health tasks through fine-tuning. To enhance their performance on specific tasks or within particular domains, both unimodal and multimodal language models can undergo fine-tuning [90]. This is a process in which a pre-trained model is further trained on a smaller, task-specific dataset. While large-scale pre-training equips models with broad linguistic and perceptual capabilities, fine-tuning allows for specialization by adapting the model’s parameters to the statistical characteristics and objectives of the target application. In some cases, smaller LLMs that have been carefully fine-tuned on domain-relevant data can outperform much larger general-purpose models [91], particularly when computational efficiency, data privacy, or deployment constraints are important considerations. For example, a lightweight model fine-tuned on therapy transcripts and synchronized voice tone or facial expression data might produce more contextually appropriate and emotionally attuned responses in a mental health setting than a general-purpose model that lacks exposure to such multimodal interactions [74,75]. Fine-tuning thus serves not only to improve performance but also to support the development of specialized, resource-efficient models tailored for high-stakes applications such as mental health care.

## Challenges

MLLMs offer promising opportunities for advancing mental health care, but their adoption also raises critical challenges that must be carefully addressed. In this section, we highlight current key concerns.

### Accuracy and reliability

In multimodal mental health modelling, accuracy depends not only on language understanding but also on the correct fusion and temporal synchronization of heterogeneous signals. Small errors in temporal alignment between speech, facial cues, and physiological responses can yield spurious correlations or misattributed emotional states. Moreover, modality-specific noise (e.g., lighting variation in video, background noise in audio, motion artifacts in wearables) propagates through embedding layers and can distort downstream reasoning. Ensuring the accuracy and reliability of MLLMs is therefore a critical prerequisite for their safe integration into mental health care. While these models show promise in generating plausible and empathetic responses, their clinical performance must be rigorously validated through systematic testing, benchmarking, and real-world trials. Without such validation, there is a risk that models may produce outputs that are factually incorrect, contextually inappropriate, or potentially harmful hallucinations [92]. To address this, recent studies have begun evaluating the performance of LLMs and MLLMs in psychological tasks under experimental conditions [13,61]. These efforts represent important first steps, but broader, more diverse, and clinically grounded assessments are needed to establish trust and ensure safe deployment in high-stakes mental health contexts.

### Bias and fairness

Biased interpretations of multimodal signals may reinforce stereotypes or misclassify distress among underrepresented populations. MLLMs raise serious concerns about bias and fairness [93]. These models are trained on large-scale multimodal datasets that often encode existing societal and cultural biases, including those related to race, gender, age, and mental health stigma [94–96]. Textual data may reflect biased language in how mental health conditions are described, while visual data may underrepresent marginalized groups, resulting in uneven model performance across populations. In multimodal systems, these biases can be compounded, further amplifying disparities. For example, biased models may produce higher-quality outputs for certain demographic groups while misrepresenting or oversimplifying the mental health experiences of others [97]. This not only risks unequal access to support but may also reinforce harmful stereotypes. Addressing these issues requires deliberate efforts to curate diverse and representative training data, incorporate input from a wide range of stakeholders, and ensure transparency about model limitations [98]. Without these safeguards, MLLMs risk perpetuating the very inequities they aim to alleviate.

### User trust and acceptance

The successful integration of MLLMs in mental health care depends heavily on user trust and acceptance, both from clinicians and patients. Mental health professionals need to feel confident that these tools support, rather than undermine, their clinical judgment, while patients must trust the system enough to engage meaningfully and disclose sensitive information. Building this trust requires not only high-quality and ethical model behaviour, but also interpretability and explainability. However, MLLMs, which are based on deep learning, often behave as opaque “black boxes”, making it difficult to trace which modality or temporal window drove an output (e.g., a prediction or a therapeutic suggestion). This opacity undermines confidence in automated recommendations. When users can understand how a model arrives at its outputs, they are more likely to evaluate its reliability and limitations appropriately. Recent studies have shown that incorporating explainability mechanisms can positively influence trust in mental health applications of AI [99]. As MLLMs continue to evolve, ensuring transparent, interpretable interactions will be critical for fostering informed trust and sustained adoption.

### Regulatory and ethical issues

The use of MLLMs in mental health care introduces complex regulatory and ethical challenges that current oversight frameworks are not fully equipped to address. MLLMs, by processing and generating outputs across multiple data types, raise unique concerns around data protection, transparency, and accountability. Yet, as noted in recent policy reviews [100], these systems often operate outside existing regulatory classifications, such as those governing medical devices, which creates uncertainty regarding their legal status and the responsibilities of developers and clinicians. Ethical evaluations have further highlighted critical risks, including the potential for harm due to misinformation, breaches of privacy, opaque decision-making, and the erosion of human autonomy in therapeutic contexts [4]. As such, responsible deployment of MLLMs requires not only alignment with evolving regulations but also proactive ethical design practices, including transparent communication of model limitations, rigorous testing, and meaningful involvement of diverse stakeholders [101].

### Deployment and interoperability

Integrating MLLMs into mental health workflows presents deployment barriers because mental health services may be delivered in resource-constrained or community-based settings rather than highly digitized environments. Deployment of MLLMs in clinical environments demands rapid inference on constrained hardware (e.g., mobile devices, legacy workstations, point-of-care kiosks), yet leading models often require substantial computational resources, precluding use in low-resource settings [102]. Employing model compression methods (e.g., parameter quantization, model pruning, low-rank decomposition, knowledge distillation) [103] and fine-tuning model variants can mitigate latency spikes and reduce computational load. Also, like other clinical decision-support tools, MLLMs must integrate seamlessly with existing EHR systems and clinical workflows to maximize their utility [14]. Achieving this integration demands close collaboration among clinicians, researchers, and health-informatics specialists [104].

## Conclusion

In this review, we aimed to equip researchers, clinicians, and developers with a comprehensive understanding of the current capabilities, limitations, and future directions for the deployment of MLLMs in mental health contexts. We synthesized emerging empirical and conceptual evidence on the use of generative MLLMs in mental health care, highlighting their potential to enhance diagnostic support, therapy, monitoring, and education through richer, context-aware, and personalized interactions. While existing applications demonstrate promising technical feasibility and therapeutic potential, the empirical foundation of this field remains preliminary, and critical challenges persist, particularly regarding clinical validation, fairness, user trust, and regulatory oversight. Addressing these challenges will be essential to ensure that MLLMs evolve into safe, effective, and ethically aligned tools. Ultimately, with continued research and responsible development, MLLMs have the potential to augment mental health care delivery in ways that are both human-centred and technologically innovative.
